# Supplementary material for: Multimodal cell tracking from systemic administration to tumour growth by combining gold nanorods and reporter genes
Source: eLife. 2018 Jun 27;7:e33140. doi: 10.7554/eLife.33140 (PMC6021173; doi:10.7554/eLife.33140)
Supplement: Figure 5—source data 1. — A similar percentage of decrease in total BLI signal intensity was observed in mice treated with GNR-unlabelled cells as in mice treated with GNR-860-labelled cells, suggesting that GNRs did not affect cell death. [file elife-33140-fig5-data1.docx]

|  | Total BLI  (day 0)  (ρ · s^-1^ · cm^-2^ · sr^-1^) | Total BLI  (day 1)  (ρ · s^-1^ · cm^-2^ · sr^-1^) | remaining signal  (%) |
| --- | --- | --- | --- |
| control-1 | 7,01E+07 | 1,00E+07 | 14,3 |
| control-2 | 3,68E+07 | 1,10E+07 | 29,9 |
|  |  |  |  |
| GNR 860-1 | 5,60E+07 | 8,62E+06 | 15,4 |
| GNR 860-2 | 3,56E+07 | 8,03E+06 | 22,6 |
| GNR-860-3 | 1,75E+07 | 2,75E+06 | 15,7 |

**Figure 5-Source Data 1. Loss in BLI signal intensity within 24 hours after injection.** A similar percentage of decrease in total BLI signal intensity was observed in mice treated with GNR-unlabelled cells as in mice treated with GNR-860-labelled cells, suggesting that GNRs did not affect cell death.
