# Supplementary material for: Multimodal cell tracking from systemic administration to tumour growth by combining gold nanorods and reporter genes
Source: eLife. 2018 Jun 27;7:e33140. doi: 10.7554/eLife.33140 (PMC6021173; doi:10.7554/eLife.33140)
Supplement: Transparent reporting form [file elife-33140-transrepform.pdf]

eLIFE
1st Floor
24 Hills Road
Cambridge CB2 1JP, UK
P 01223 855340
W elifesciences.org
T @elife

eLife's transparent reporting form

We encourage authors to provide detailed information *within their submission* to facilitate the interpretation and replication of experiments. Authors can upload supporting documentation to indicate the use of appropriate reporting guidelines for health-related research (see EQUATOR Network), life science research (see the BioSharing Information Resource), or the ARRIVE guidelines for reporting work involving animal research. Where applicable, authors should refer to any relevant reporting standards documents in this form.

If you have any questions, please consult our Journal Policies and/or contact us: editorial@elifesciences.org.

Sample-size estimation

- You should state whether an appropriate sample size was computed when the study was being designed
- You should state the statistical method of sample size computation and any required assumptions
- If no explicit power analysis was used, you should describe how you decided what sample (replicate) size (number) to use

Please outline where this information can be found within the submission (e.g., sections or figure legends), or explain why this information doesn't apply to your submission:

Sample-size estimations do not apply to our manuscript, since our study does not include quantitative datasets that require statistical analysis for significant differences. We are not comparing treatment groups or different conditions. In our 'tools and resources' type of manuscript, we describe and characterize novel tools for cell labelling and tracking *in vivo*. The data are mostly of a qualitative nature. We test labelled cells in several mice, each of which constitutes an individual example to proof the quality of the tools. The original source data for these have been deposited in the research data repository Zenodo (https://zenodo.org/record/1021607) (doi: 10.5281/zenodo.1021607) for open access. An overview of animals used in our study is provided in Table 1 of the manuscript (Materials and Methods section).

Replicates

- You should report how often each experiment was performed
- You should include a definition of biological versus technical replication
- The data obtained should be provided and sufficient information should be provided to indicate the number of independent biological and/or technical replicates
- If you encountered any outliers, you should describe how these were handled
- Criteria for exclusion/inclusion of data should be clearly stated
- High-throughput sequence data should be uploaded before submission, with a private link for reviewers provided (these are available from both GEO and ArrayExpress)

Please outline where this information can be found within the submission (e.g., sections or figure legends), or explain why this information doesn't apply to your submission:

eLife Sciences Publications, Ltd is a limited liability non-profit non-stock corporation incorporated in the State of Delaware, USA, with company number 5030732, and is registered in the UK with company number FC030576 and branch number BR015634 at the address 1st Floor, 24 Hills Road, Cambridge CB2 1JP | August 2014

We provide an overview of replicates of animal experiments in Table 1 of our manuscript (located in Material and Methods). Each mouse serves as an individual example in the collection of qualitative data (i.e. a biological replicates). For each animal experiment, cells were newly labelled with gold-nanorods (biological repeats). The cells have been labelled genetically via Lentivirus transfection, FACS sorted to obtain a population with high expression levels of reporter genes, and then maintained as a stock in culture for labelling with GNRs and associated experiments. The genetically labelled stock of cells, from which aliquots were used in individual experiment, can be considered as technical repeats.
The cell viability shown in Figure 1e was assessed in three biological replicates as described in the figure legend and in the Methods section.

Statistical reporting

- Statistical analysis methods should be described and justified
- Raw data should be presented in figures whenever informative to do so (typically when N per group is less than 10)
- For each experiment, you should identify the statistical tests used, exact values of N, definitions of center, methods of multiple test correction, and dispersion and precision measures (e.g., mean, median, SD, SEM, confidence intervals; and, for the major substantive results, a measure of effect size (e.g., Pearson's r, Cohen's d)
- Report exact p-values wherever possible alongside the summary statistics and 95% confidence intervals. These should be reported for all key questions and not only when the p-value is less than 0.05.

Please outline where this information can be found within the submission (e.g., sections or figure legends), or explain why this information doesn't apply to your submission:

We do not apply statistical reporting in our 'tools and resources' type of manuscript. The data are mostly of a qualitative nature, describing the characteristics of the imaging tools in individual case studies (animals). Our study does not include quantitative datasets that require statistical analysis and comparison for significant differences. We are not comparing treatment groups or different conditions quantitatively.

(For large datasets, or papers with a very large number of statistical tests, you may upload a single table file with tests, Ns, etc., with reference to sections in the manuscript.)

Group allocation

- Indicate how samples were allocated into experimental groups (in the case of clinical studies, please specify allocation to treatment method); if randomization was used, please also state if restricted randomization was applied
- Indicate if masking was used during group allocation, data collection and/or data analysis

Please outline where this information can be found within the submission (e.g., sections or figure legends), or explain why this information doesn't apply to your submission:

The mice used in our experiments were randomly allocated into groups receiving differently labelled cells (control, GNR-710, GNR-860; Table 1 in Materials and Methods). Randomization was not restricted. Masking was not used, since mice would have been identifiable in any case, according to the photoacoustic imaging signals they provide.

Additional data files ("source data")

- We encourage you to upload relevant additional data files, such as numerical data that are represented as a graph in a figure, or as a summary table
- Where provided, these should be in the most useful format, and they can be uploaded as "Source data" files linked to a main figure or table
- Include model definition files including the full list of parameters used
- Include code used for data analysis (e.g., R, MatLab)
- Avoid stating that data files are "available upon request"

Please indicate the figures or tables for which source data files have been provided:

Source data for our animal experiments have been deposited in the research data repository Zenodo (https://zenodo.org/record/1021607) (doi: 10.5281/zenodo.1021607) for open access, in line with the ARRIVE guidelines on reporting of animal experiments.
An overview of animals used in our study is provided in Table 1 of the manuscript (Materials and Methods section). These source data are relevant for Figures 3 – 8 of the manuscript.
